# Immune Characteristics in Biliary Atresia Based on Immune Genes and Immune Cell Infiltration

**DOI:** 10.3389/fped.2022.902571

**Published:** 2022-05-23

**Authors:** Chenyu Yang, Huiwu Xing, Bingqian Tan, Mingman Zhang

**Affiliations:** ^1^Department of Hepatobiliary Surgery Children's Hospital of Chongqing Medical University, Chongqing Key Laboratory of Pediatrics, National Clinical Research Center for Child Health and Disorders, Ministry of Education Key Laboratory of Child Development and Disorders, Chongqing, China; ^2^Chongqing Higher Institution Engineering Research Center of Children's Medical Big Data Intelligent Application, Chongqing, China

**Keywords:** biliary atresia, immune genes, immune cell infiltration, inflammation, liver - fibrosis and - cirrhosis

## Abstract

**Background:**

Biliary atresia (BA) is a serious biliary disease in infancy. Jaundice is the most visual and prominent symptom, and it mainly involves bile duct cells leading to the loss of intrahepatic and extrahepatic bile ducts. If left untreated, it will eventually progress to liver cirrhosis. The pathogenesis of BA is not clear, and it is now generally accepted that BA is an autoimmune disease. However, few studies have revealed the infiltration of immune cells in the liver of BA from a global perspective. We used liver tissue sequencing data to predict the infiltration and relative content of immune cells in BA.

**Methods:**

The BA datasets GSE46960, GSE15235, and GSE84044, and patient information were downloaded from the Gene Expression Omnibus (GEO) database. After batch normalization, the differentially expressed immune genes (DE-IGs) in BA liver, normal liver, and hepatitis B liver were analyzed with the cut-off value of |log_2_fold change (log_2_FC)| >1 and false discovery rate (FDR) <0.05. CIBERSORT software was used to predict the proportions of 22 immune cells in all samples of the datasets.

**Results:**

73 DE-IGs have been screened out between BA and normal tissue; among them, 20 genes were highly expressed and another 53 were expressed at a low level. A total of 30 DE-IGs existed between inflammation and fibrosis livers of BA, and all of them were expressed at low levels in fibrosis livers of BA. In GO term analysis, these DE-IGs were mainly associated with the MHC protein complex, cytokine, chemokine activity, and MHC-II receptor activity. In KEGG pathway analysis, the DE-IGs were mainly enriched in pathways of Th1 and Th2 cell differentiation, Th17 cell differentiation, IL-17 signaling pathway, Toll–like receptor signaling pathway, TNF signaling pathway, and autoimmune diseases. There were significant differences in immune infiltration among different pathological types of BA, and there were also obvious differences in immune infiltration of hepatitis B as a disease control of BA.

**Conclusion:**

Based on immune genes and immune cell infiltration, this study reveals the immune characteristics of BA from a global point of view, which provides a new perspective for understanding the pathogenesis of BA and provides a direction for the diagnosis and treatment of BA.

## Introduction

Biliary atresia (BA) is a fibroinflammatory obliterative cholangiopathy peculiar to infants with the characteristics of obstruction of extra- and intrahepatic biliary tree, finally leading to cirrhosis and liver failure (1–3). Asian newborns are more likely to suffer from BA (1). In Asia, the overall incidence of BA per 10,000 live births was ~1.06–1.8 (4, 5). However, the incidence was lower in Europe and the US, 0.52–0.66 per 10,000 live births (6–8). The pathogenesis of BA is still unclear and it is believed that different factors, including genetic, infectious, inflammatory, and even toxic insults, contribute to the initiation and development of BA. Kasai hepatic portoenterostomy (HPE) and liver transplantation (LT) are the main and classical treatments of BA (1, 2). If left untreated, persistent cholestasis will lead to progressive liver cirrhosis and eventually death at the age of 2 years (1).

Although the etiological factors of BA are not clear, it is certain that the immune system plays an important role in the development of the disease and affects the outcome, as both innate immunity and specific immunity are involved in BA (3). Biliary epithelial cells (cholangiocytes) initiate innate immunity and activate inflammation by recognizing the pathogen-associated molecular patterns (PAMPs) of microorganisms through toll-like receptors (TLRs) (9, 10). Other innate immune cells, such as macrophages, dendritic cells (DCs), and natural killer (NK), are also associated with immune and inflammatory responses (3, 9, 11). Specific immunity also affects the occurrence and development of diseases. Th1 cells can create a pro-inflammatory microenvironment by producing IFN-γ, which activates CD8^+^ T cells and B cells to perpetuate biliary tree damage (12, 13). Therefore, the immune system mediates chronic inflammation, duct proliferation, and fibrosis in post-obstruction.

**Figure 1:**
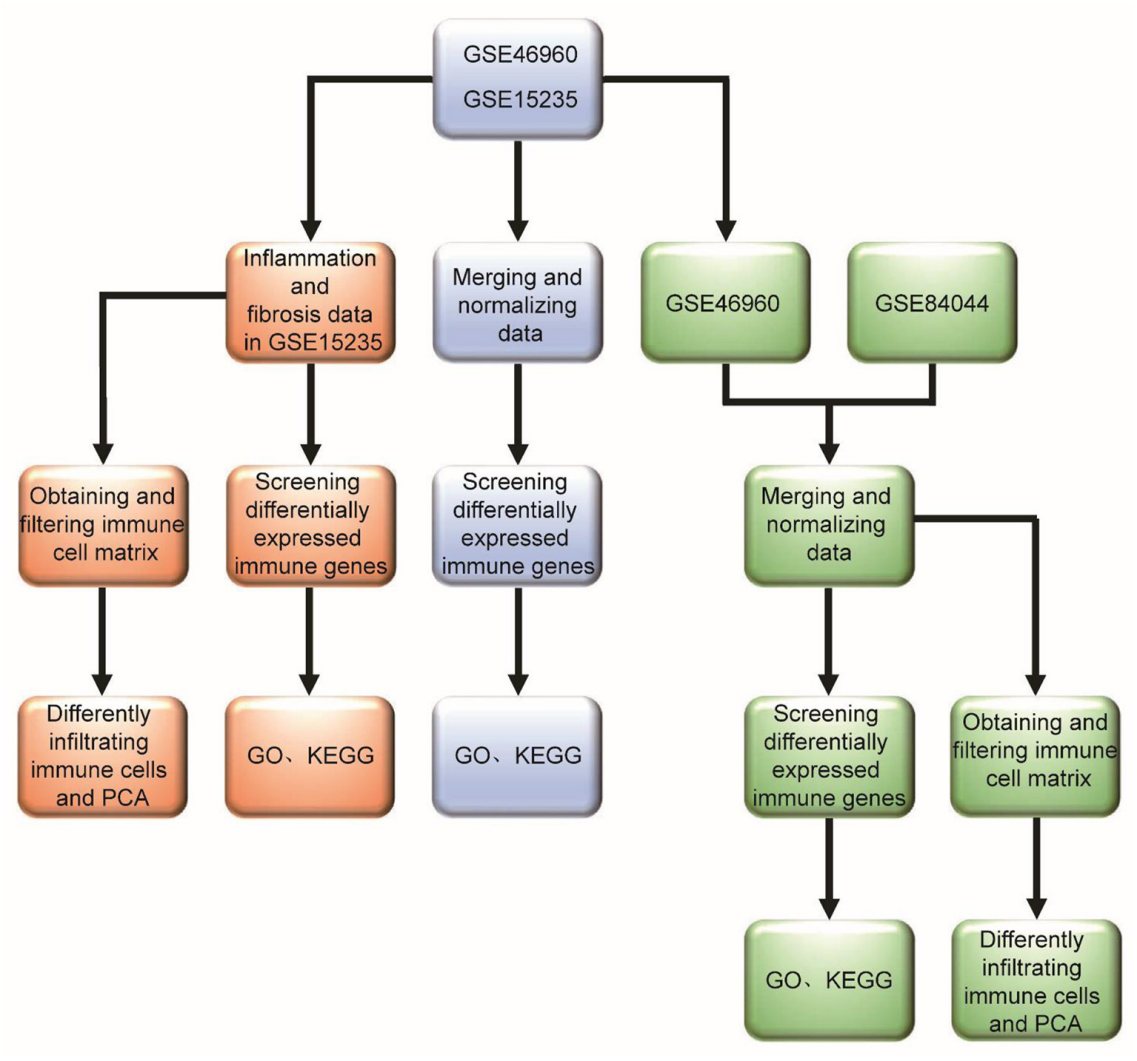
Research flowchart of this study.

There have been research reports that molecular profiling could help diagnose inflammation or fibrosis in most BA livers (14). The signatures may be associated with the staging of disease at the diagnosis and could forecast clinical outcomes. However, many studies mostly focus on one immune cell associated with BA. Therefore, we hope to find the differences between BA and normal livers, and the differences between inflammatory infiltration and liver cirrhosis in BA from the point of view of immune-related genes and immune cell infiltration. Some viruses have been demonstrated to be associated with BA, but interestingly, a few studies report that Hepadnaviridae, such as Hepatitis B virus and Hepatitis C virus (HBV and HCV), could cause BA, which can be transmitted vertically from mothers to babies. Moreover, the humoral immunogenicity of children with BA was insufficient after vaccination with recombinant HBV vaccine (15). Therefore, we raise the question that rotavirus and cytomegalovirus can cause BA, while Hepatoviridae are difficult, but they have an unknown underlying link in the BA liver, so what is the difference in immune response between these types of viral infections? Our research flowchart is described in Figure 1.

## Methods

### Data Downloaded and Batch Normalization

The patient expression profile matrix and clinical information of the datasets GSE46960, GSE15235, and GSE84044 were downloaded from the GEO database (https://www.ncbi.nlm.nih.gov/geo/) (14, 16, 17). When two datasets needed to be analyzed together, it was necessary to perform preprocessing, data integration, normalization, and batch-effect correction in advance.

### Pathological Typing of the BA Liver

The pathological types of BA were grouped according to the grades of inflammation and the stage of fibrosis. The grades of inflammation of the livers were observed microscopically after HE staining and could be classified into four grades, Grade 0: no inflammation; Grade 1: mild portal inflammation; Grade 2: portal expansion prominent inflammation in <50% portal tracts; Grade 3 portal expansion brisk inflammation in >50% portal tracts. Masson staining followed by microscopic observation of the liver fibrosis stage can be divided into four stages, Stage 0: no fibrosis; Stage 1: mild portal fibrosis; Stage 2: portal fibrosis (expansion + bridging in <50% portal tracts); Stage 3: portal fibrosis (expansion + bridging in >50% portal tracts or regenerative nodule). Pathological type of inflammation based on inflammation minus fibrosis scores ≥2 and pathological type of fibrosis based on inflammation minus fibrosis scores ≤ -2. For more detailed grouping methods and information, please refer to Reference 18.

### Extracting DE-IGs and Functional Enrichment Analysis

The differentially expressed mRNAs were analyzed by the limma package in R software (https://www.r-project.org/) with the cut-off value of |log_2_fold change (log_2_FC)| >1 and false discovery rate (FDR) <0.05. The differentially expressed mRNAs were visualized in volcano plots and heatmaps in R. The immune gene list was downloaded from ImmPort (https://www.immport.org/). The differentially expressed immune genes (DE-IGs) were filtered from the differentially expressed mRNAs. DE-IGs functional enrichment analyses were performed to identify the major biological function, and the Gene Ontology (GO) and Kyoto Encyclopedia of Genes and Genomes (KEGG) enrichment analyses were performed. The DOSE, clusterProfiler, and enrichplot packages were used to visualize the enrichment terms.

### Immune Cell Infiltration Analysis

CIBERSORT software (http://CIBERSORT.stanford.edu/) was used to predict the proportions of 22 immune cells in all samples of the datasets. The CIBERSORT package in R was used to assess the abundance of the 22 immune cell types in the liver. The barplot and heatmap reflected the relative content of 22 immune cells in each sample. The correlation heatmap analyzed the correlation between immune cells and visualized them. The violin plot revealed the difference in the content of immune cells among different groups. Principal component analysis (PCA) was used to investigate the immune cell infiltration relationship between individuals.

## Results

### GO and KEGG Analysis of DE-IGs Between Normal Liver and BA

**Figure 2:**
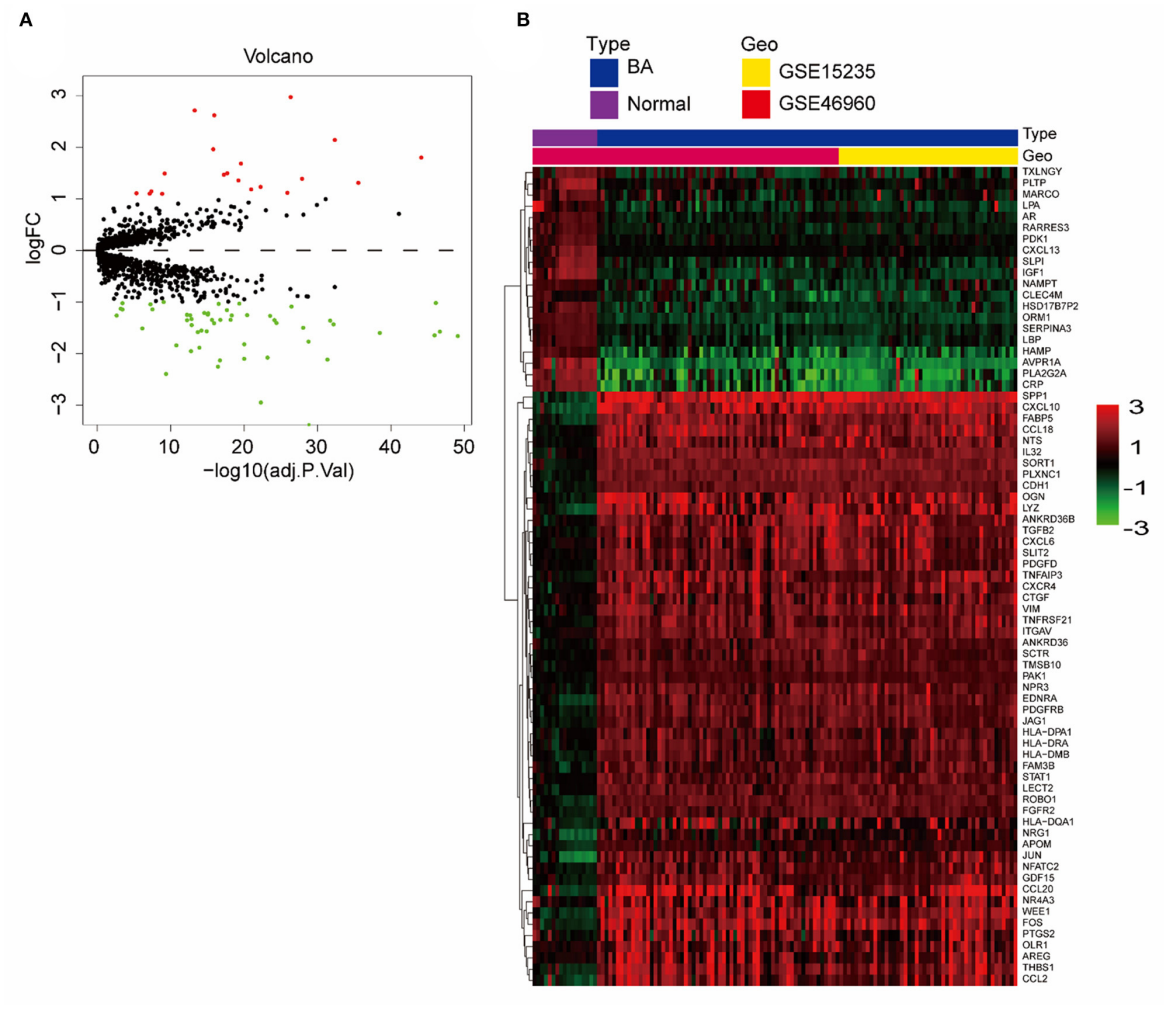
DE-IGs between normal liver and BA. **(A)** The volcano plot of 73 DE-IGs between normal liver and BA. The red plots represent upregulated genes and the green plots represent downregulated genes. **(B)** The heatmap reflects the relative expression of DE-IGs in different individuals and the overall difference between the two groups.

We merged and normalized the two datasets, GSE46960 and GSE15235, to generate a new dataset including, 111 BA and 17 donor livers (10 adults and seven children). The donor livers were identified as normal livers to be the control group of BAs. Comparing the expression between BA livers and normal livers, 73 DE-IGs were screened out, 20 genes were highly expressed, and another 53 were lowly expressed (Figure 2A). The specific expression trends of these genes in the two groups were described in the heatmap, and the clustering revealed significant differences in the gene expression profiles of normal and BA livers, as well as consistency in the gene expression profiles of individuals within the group (Figure 2B). This result also showed that the immune gene expression was not significantly different between the livers of children and adults.

**Figure 3:**
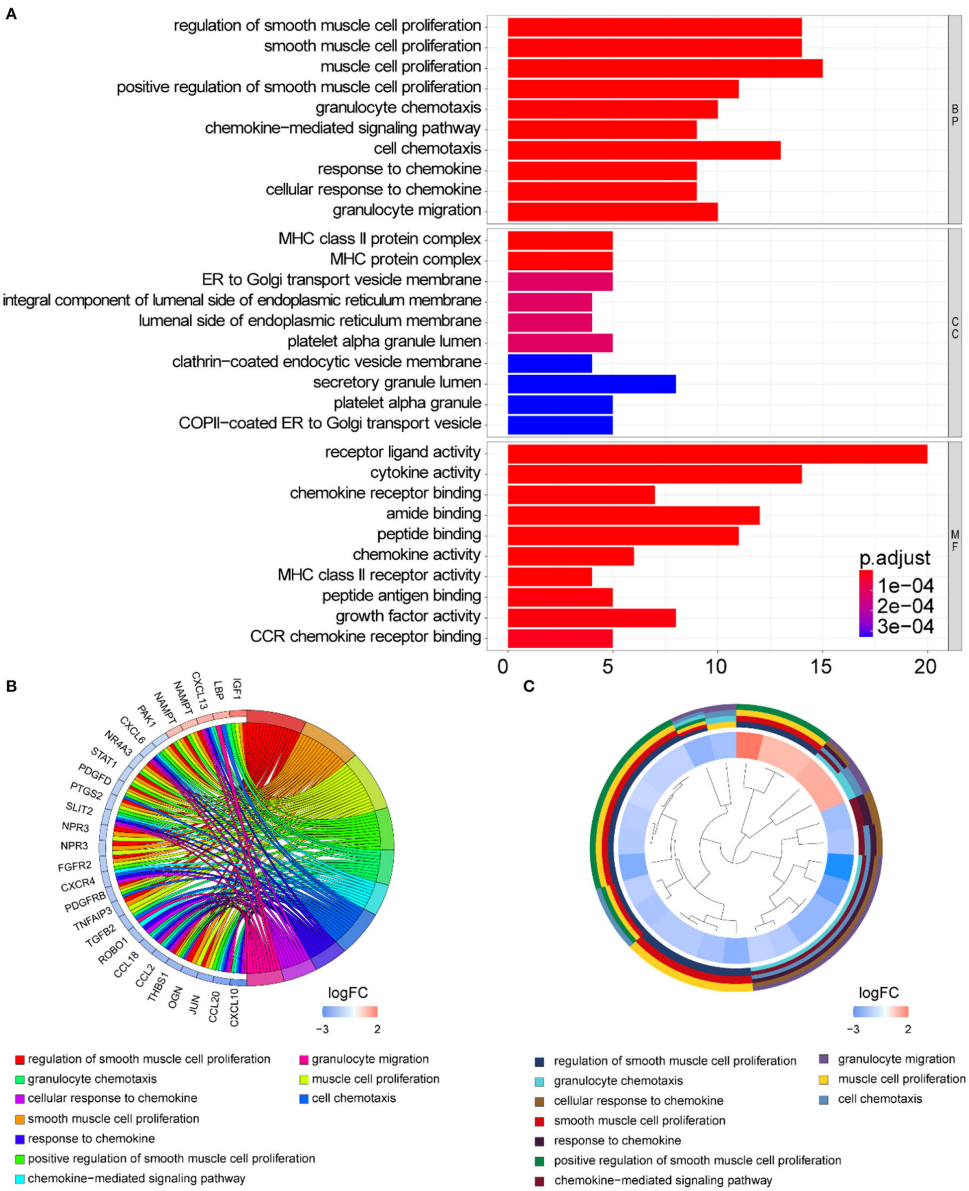
GO analysis of the DE-IGs between normal liver and BA. **(A)** The 10 most significantly enriched GO terms are displayed in the barplot. The length of the bar represents the number of genes enriched, and the color represents the correlation. BP, biological process; CC, cellular component; MF, molecular function. **(B)** GO chord diagram of the DE-IGs. The outer circle represents different genes and GO terms. The color depth of the outer circle of genes represents the logFC indicating the gene expression level. The internal colorful ribbon represents the different GO terms the genes enriched. **(C)** The GO cluster of the DE-IGs. The outer circle represents the GO terms the genes enriched, and the inner circle represents the logFC of each gene, with the color depth corresponding to the gene expression level.

**Figure 4:**
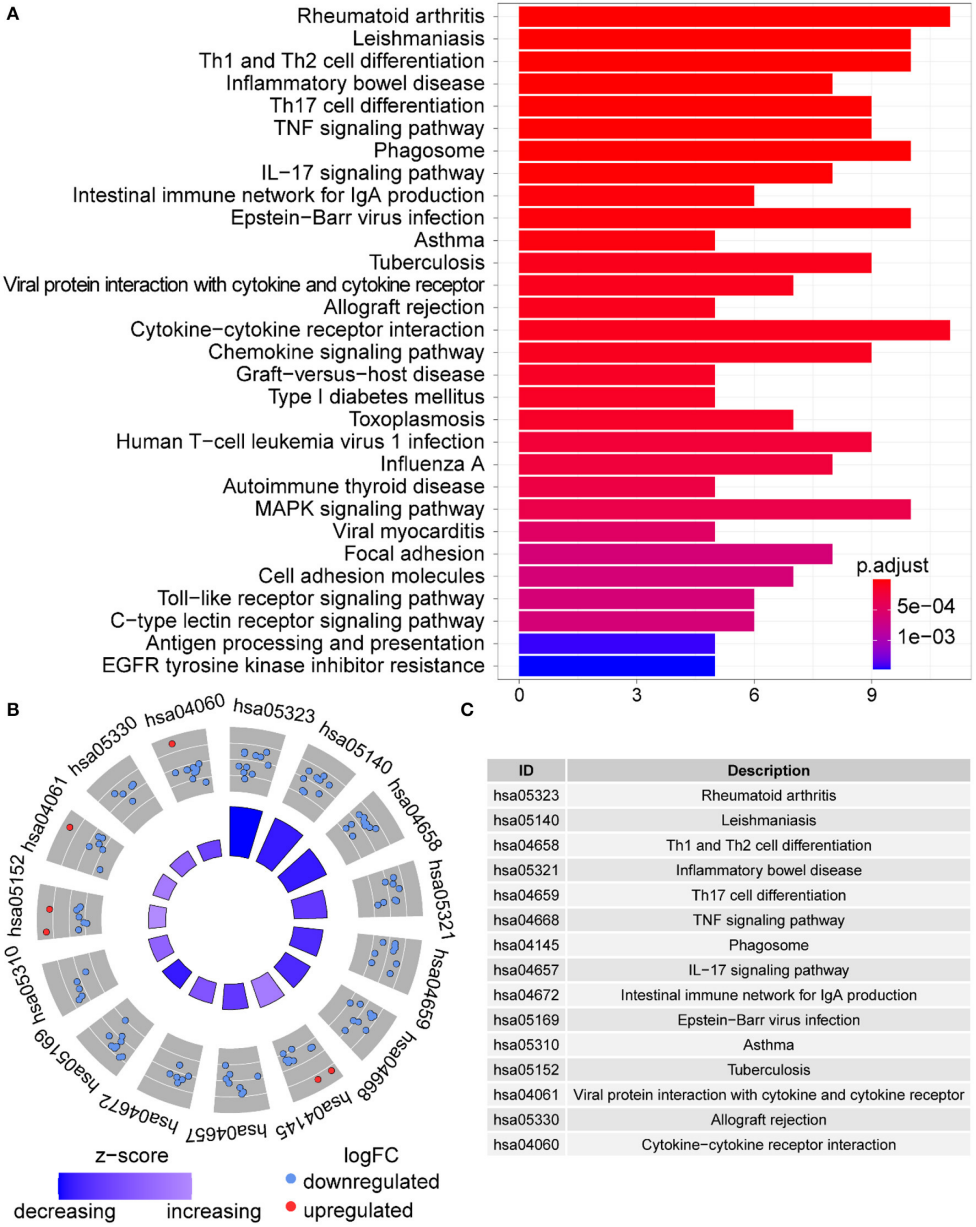
KEGG analysis of the DE-IGs between normal liver and BA. **(A)** The 30 most significantly enriched KEGG pathways are displayed in the barplot. The length of the bar represents the number of genes enriched and the color represents the correlation. **(B)** The circle of KEGG enrichment analysis. Each dot in the circle represents a gene and the outer circle reflects the enrichment of the DE-IGs in different signaling pathways. The inner circle represents the Z-score, the color depth corresponding to the Z-score. Higher Z-score indicates a higher expression of the DE-IGs in enriched pathways. **(C)** The table annotates KEGG pathways.

Functional enrichment of the 73 DE-IGs was performed by GO term (Figure 3) and KEGG pathway (Figure 4) analyses. The enriched biological process (BP) of GO related to granulocyte function obviously included granulocyte chemotaxis and migration, and immune function, such as cell chemotaxis. In the cellular component (CC), these genes were enriched in the MHC protein complex and some terms were associated with the intracellular vesicle. For molecular function (MF), these differentially expressed genes were closely related to receptor–ligand activity, of which MHC class II receptors are representative. In KEGG pathway analysis, the DE-IGs showed notable associations with the pathways in innate immunity and adaptive immunity responses, including the Toll–like receptor signaling pathway, C–type lectin receptor signaling pathway, Th1 and Th2 cell differentiation, and IL−17 signaling pathway.

### GO and KEGG Analysis of DE-IGs Between Livers With Inflammation and Fibrosis Livers in BA

**Figure 5:**
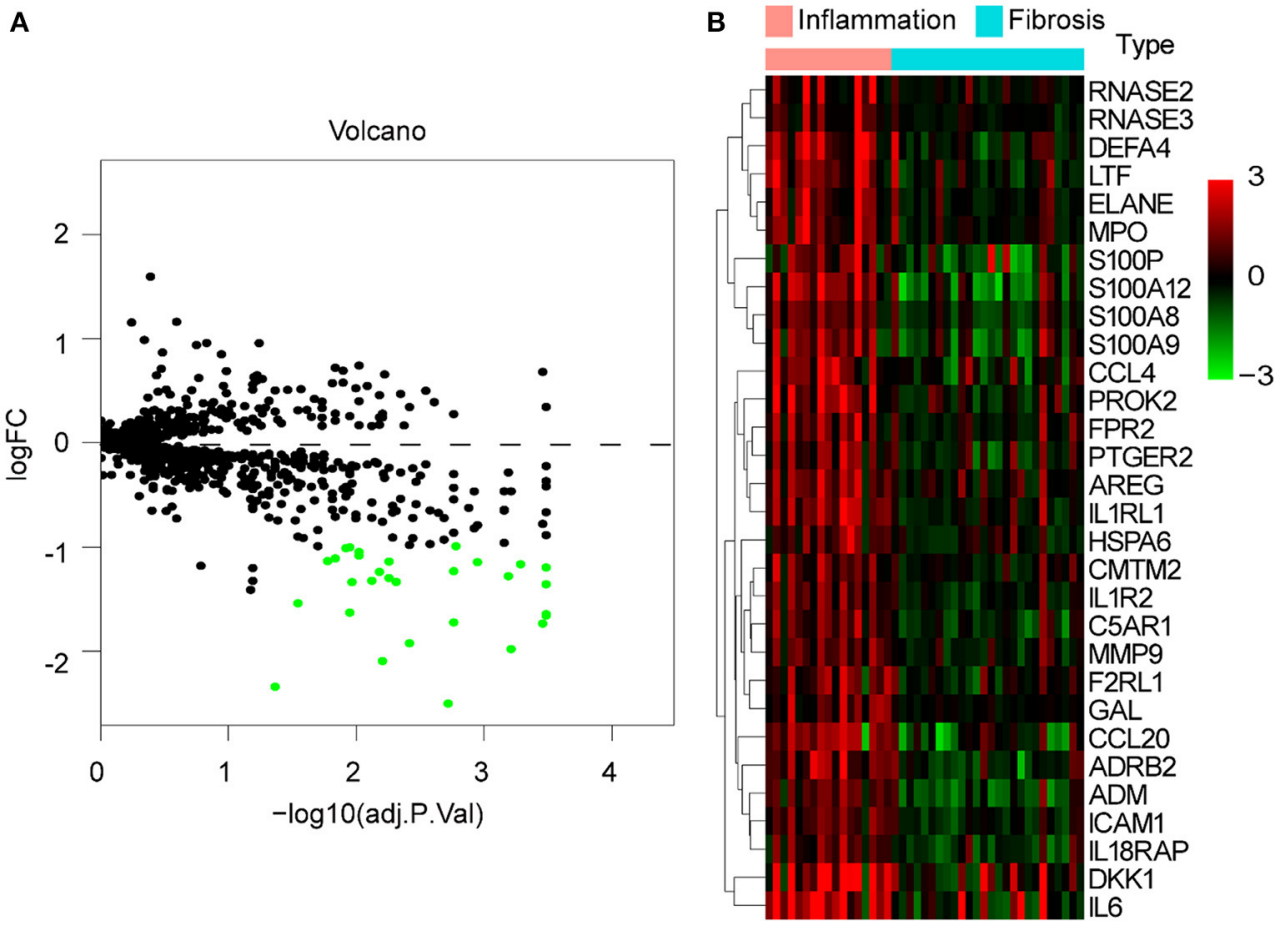
DE-IGs between inflammation and fibrotic livers of BA. **(A)** The volcano plot of 30 DE-IGs. The red plots represent upregulated genes and the green plots represent downregulated genes. **(B)** The heatmap reflects the relative expression of DE-IGs in different individuals and the overall difference between the two groups.

To explore whether there are differences in immune responses at different stages of BA development, we selected the GSE15235 dataset for this study. GSE15235 includes 47 BAs, of which 17 and 26 livers had been diagnosed with inflammation and fibrosis based on histological and molecular features, respectively, and the specific type and stage could not be determined for the remaining four affected livers (18). We observed differential expression of 30 genes out of 1,251 candidate immune genes. Unexpectedly, all of these genes were expressed at low levels in fibrotic livers (Figure 5A). The heatmap illustrated the specific expression of these 30 differentially expressed genes in each individual and reflected an overall perspective that these genes were significantly downregulated in fibrotic livers (Figure 5B).

**Figure 6:**
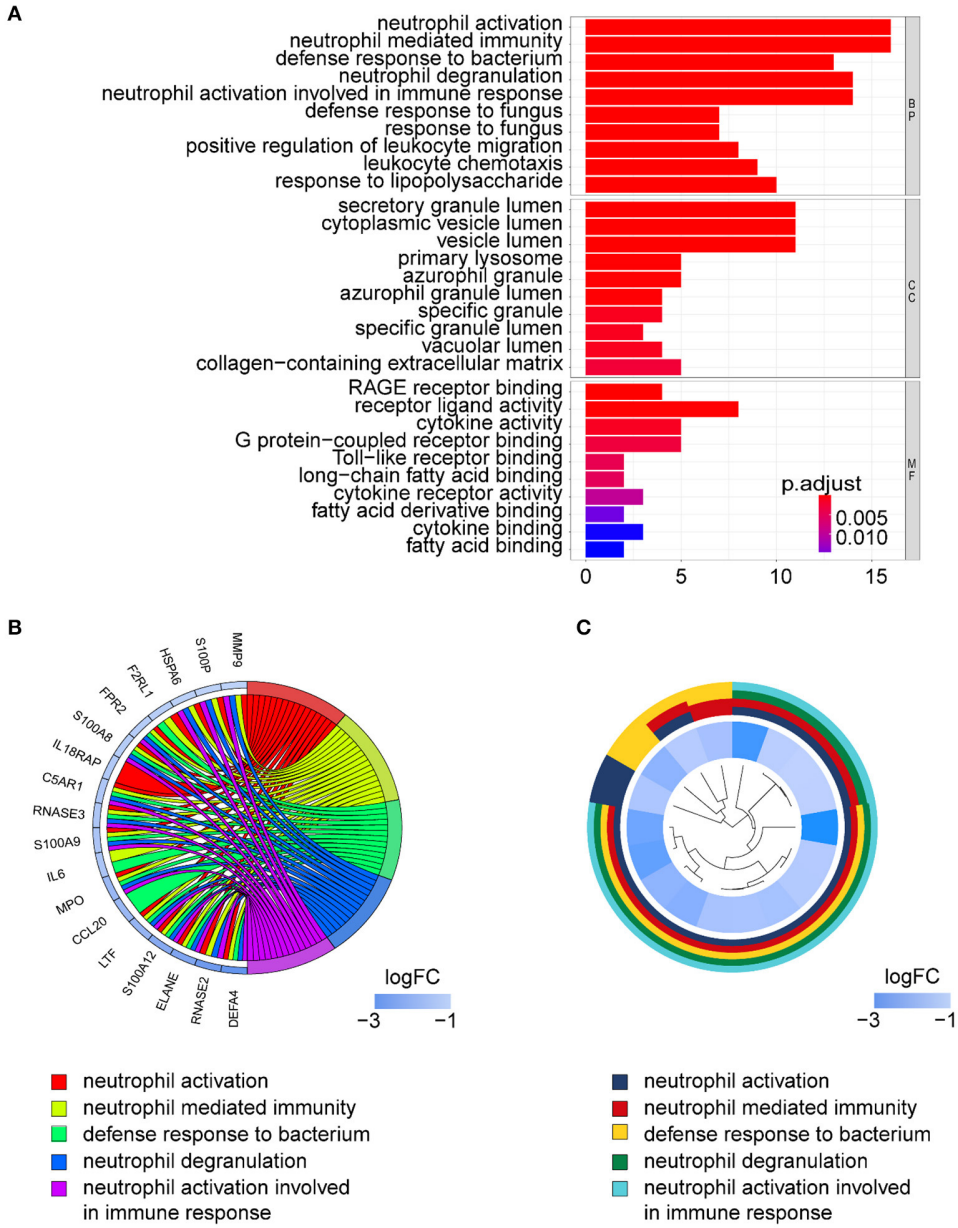
GO analysis of DE-IGs between liver with inflammation and fibrosis livers in BA. **(A)** The 10 most significantly enriched GO terms are displayed in the barplot. The length of the bar represents the number of genes enriched, and the color represents the correlation. BP, biological process; CC, cellular component; MF, molecular function. **(B)** GO chord diagram of the DE-IGs. The outer circle represents different genes and GO terms. The color depth of the outer circle of genes represents the logFC indicating the gene expression level. The internal colorful ribbon represents the different GO terms the genes enriched. **(C)** The GO cluster of the DE-IGs. The outer circle represents the GO terms that the genes enriched, and the inner circle represents the logFC of each gene, with the color depth corresponding to the gene expression level.

**Figure 7:**
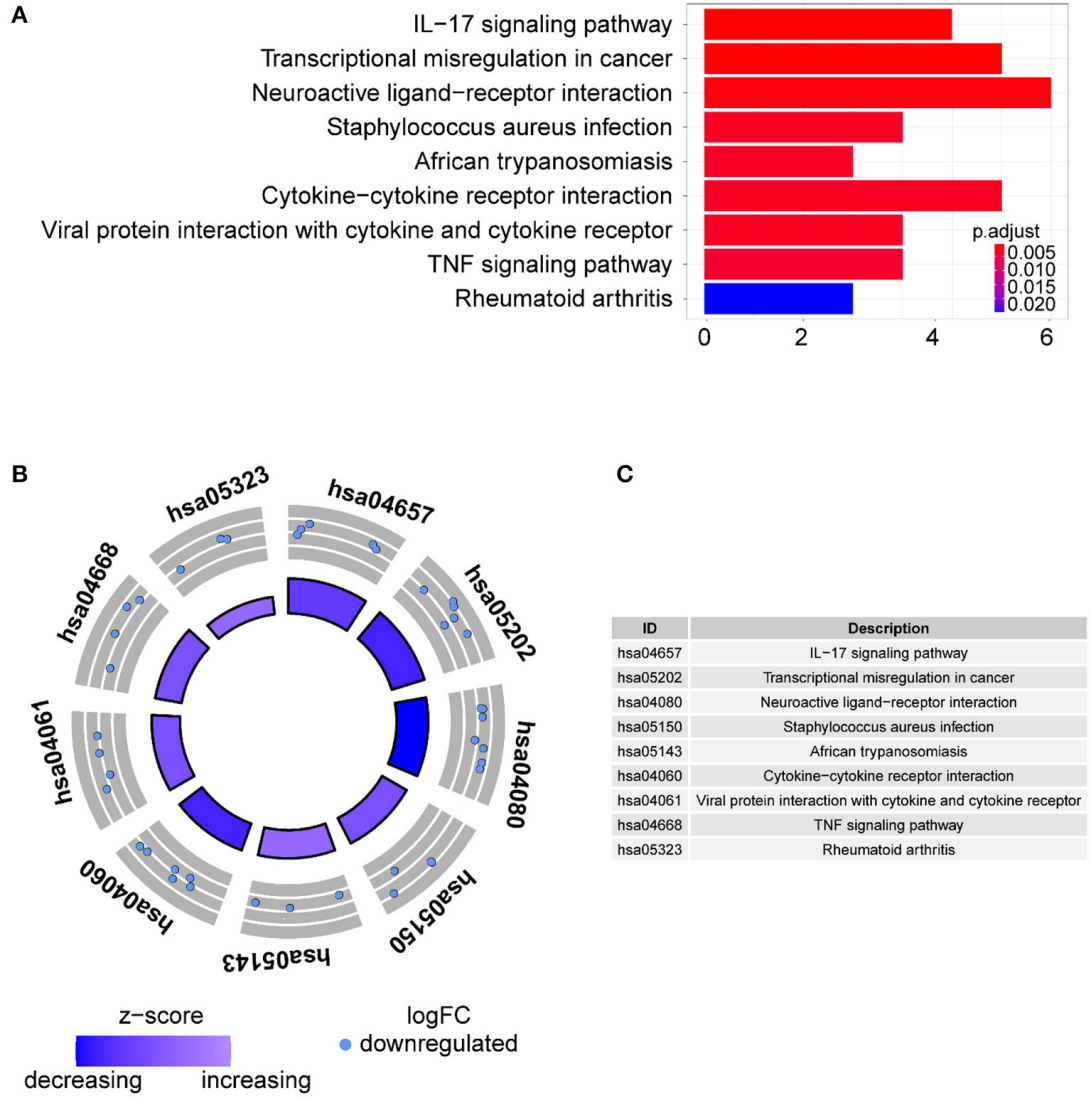
KEGG analysis of the 30 DE-IGs between inflamed and fibrotic livers of BA. **(A)** The 30 most significantly enriched KEGG pathways are displayed in the barplot. The length of the bar represents the number of genes enriched, and the color represents the correlation. **(B)** The circle of KEGG pathways enrichment analysis. Each dot in the circle represents a gene, and the outer circle reflects the enrichment of the DE-IGs in different signaling pathways. The inner circle represents the Z-score, the color depth corresponding to the Z-score. Higher Z-score indicates a higher expression of the DE-IGs in enriched pathways. **(C)** The table annotates KEGG pathways.

Gene ontology term analysis indicated that BP was significantly related to the activation and function of the neutrophil, CC was mainly related to the lysosome and enriched some intracellular vesicles and granules, and MF was associated with lipids, RAGE receptor binding, and innate immunity, such as toll-like receptor binding (Figure 6). In KEGG pathway analysis, the DE-IGs were mainly enriched in pathways of infection by some pathogenic microorganisms, viral protein interactions with cytokine and cytokine receptors, and the IL-17 signaling pathway (Figure 7).

### Immune Cells Infiltrating the Inflammation and Fibrosis Livers of BA

**Figure 8:**
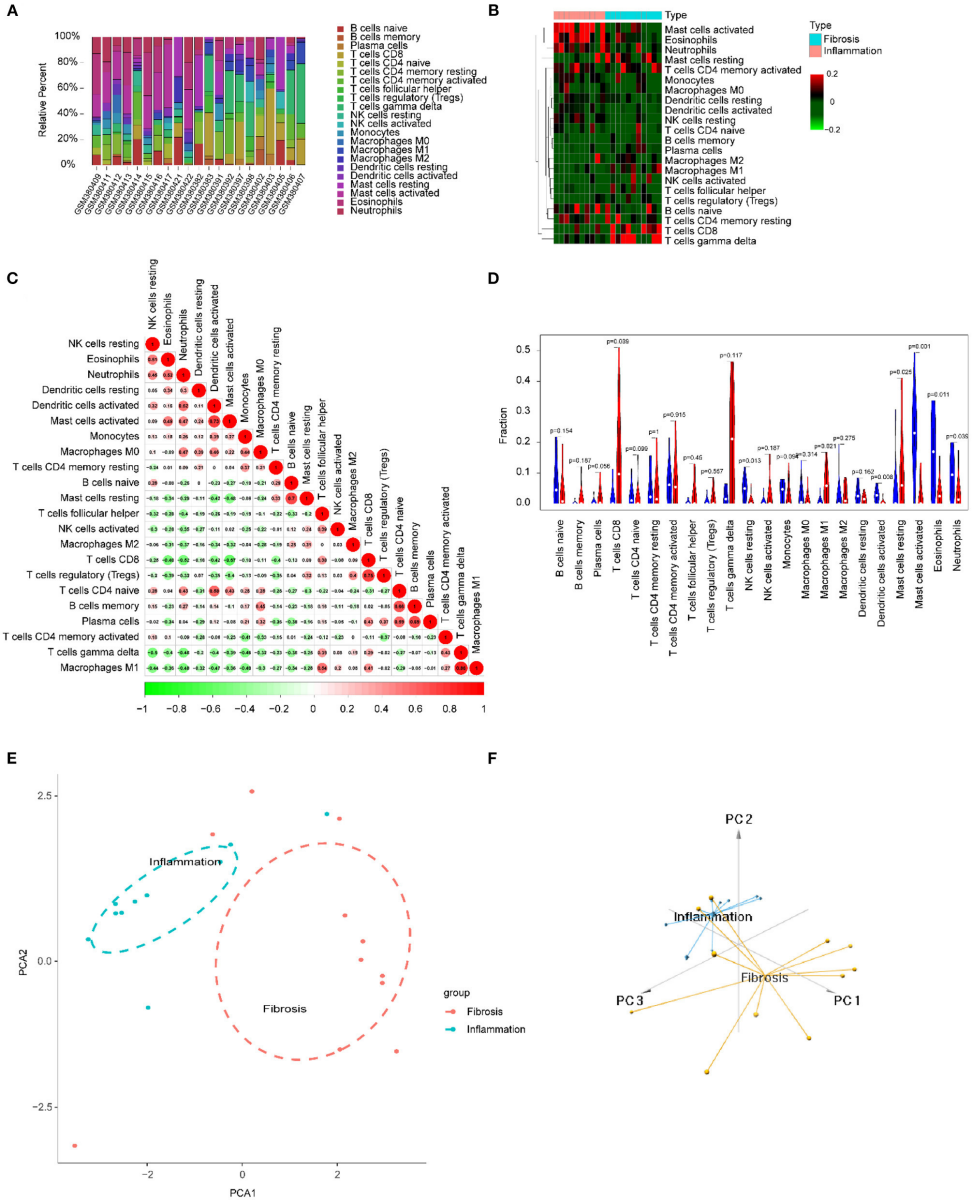
Immune cells infiltrating the livers with inflammation and fibrosis in BA. **(A)** The barplot reflects the relative content of 22 immune cells in each sample. **(B)** Heatmap of the 22 immune cell proportions. **(C)** Correlation heatmap of 22 immune cells. The size of the colored circles represents the strength of the correlation; red represents a positive correlation, and green represents a negative correlation. **(D)** Violin diagram of the proportion of 22 types of immune cells among the different groups. **(E,F)** PCA cluster plot of immune cell infiltration between inflamed and fibrotic livers of BA samples.

We focused on 22 kinds of immune cells to study their infiltrating characteristics between inflamed and fibrotic livers of BA in GSE15235. By using CIBERSORT, the gene expression matrix was transformed into the immune cell-matrix and combined with the composition and percentages of the immune cells. Based on the screening cut-off value, we obtained 21 (10 inflammatory and 11 fibrotic livers) statistically significant immune cell matrices. Analysis of the characteristics of infiltrative immune cells showed that activated mast cells, eosinophils, and neutrophils were more abundant in inflamed livers than that in fibrosis livers. In contrast, CD8^+^ T cells and γδT cells were more abundant in fibrotic livers (Figures 8A,B). The correlation heatmap indicated that activated mast cells, CD8^+^ T cells, and γδT cells were positively correlated with activated DCs, Tregs, and M1 macrophages respectively, and a moderate negative correlation existed between CD8^+^ T cells and activated mast cells (Figure 8C). The violin plots showed that fibrotic livers have more M1 macrophages and resting mast cells, whereas activated mast cells, eosinophils, and neutrophils are less frequent than in inflamed livers, indicating differences in immune cell infiltration between them (Figure 8D). PCA analysis was performed to explore the distinct distribution patterns between inflamed and fibrotic livers. The results showed that the two independent groups could be divided into two aspects based on the infiltration of the immune cells (Figures 8E,F).

### GO and KEGG Analysis of DE-IGs Between BA and Viral Hepatitis B

**Figure 9:**
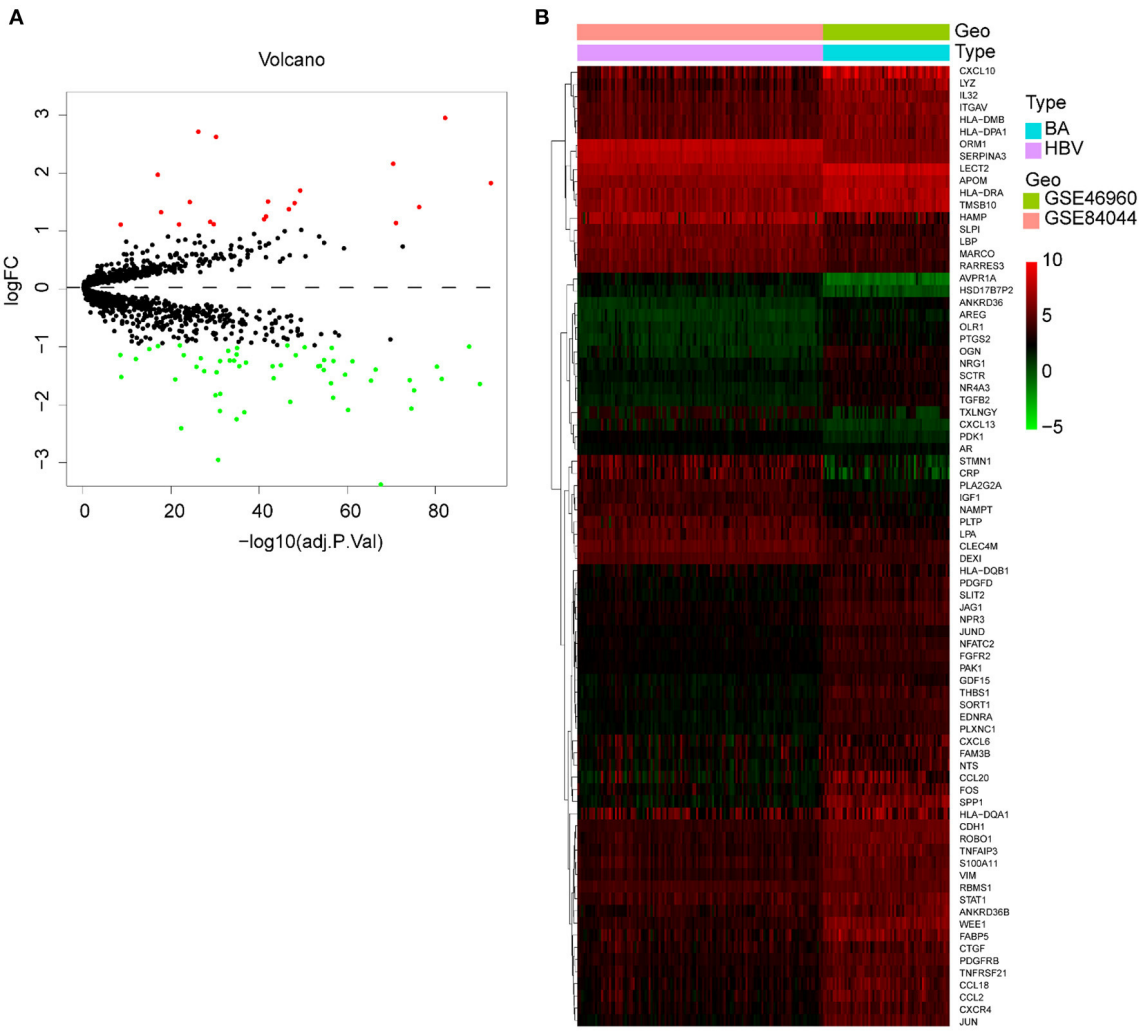
DE-IGs between BA and viral hepatitis B. **(A)** The volcano plot of 75 DE-IGs. The red plots represent upregulated genes and the green plots represent downregulated genes. **(B)** The heatmap reflects the relative expression of DE-IGs in different individuals and the overall difference between the two groups.

Since some viruses, including rotavirus and cytomegalovirus, can cause BA, but hepadnaviridae cannot, we studied the difference in immune response between these two types of virus infections. We merged and normalized the two datasets GSE46960 and GSE84044 to establish a new dataset, including 64 BA and 123 viral Hepatitis B. Seventy-five DE-IGs were screened out, of which 20 genes were upregulated and 55 genes were downregulated (Figure 9A). The heatmap described the genes' expression levels of each individual and the different trends in the two groups (Figure 9B).

**Figure 10:**
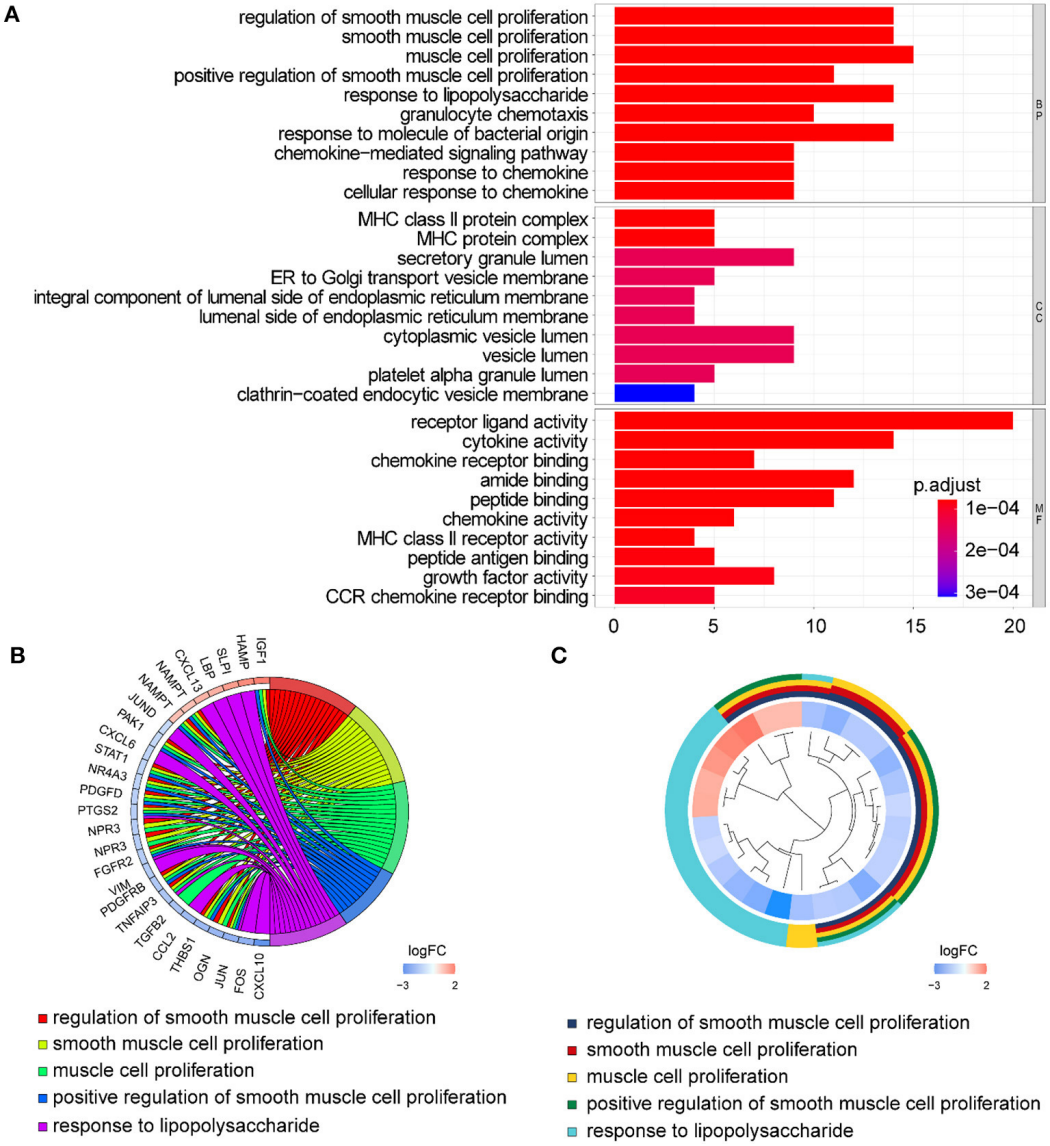
GO analysis of the 75 DE-IGs between inflamed and fibrotic livers of BA. **(A)** The 10 most significantly enriched GO terms are displayed in the barplot. The length of the bar represents the number of genes enriched and the color represents the correlation. BP, biological process; CC, cellular component; MF, molecular function. **(B)** GO chord diagram of the DE-IGs. The outer circle represents different genes and GO terms. The color depth of the outer circle of genes represents the logFC indicating gene expression level. The internal colorful ribbon represents the different GO terms the genes enriched. **(C)** The GO cluster of the DE-IGs. The outer circle represents the GO terms for the genes enriched, and the inner circle represents the logFC of each gene, with the color depth corresponding to the gene expression level.

**Figure 11:**
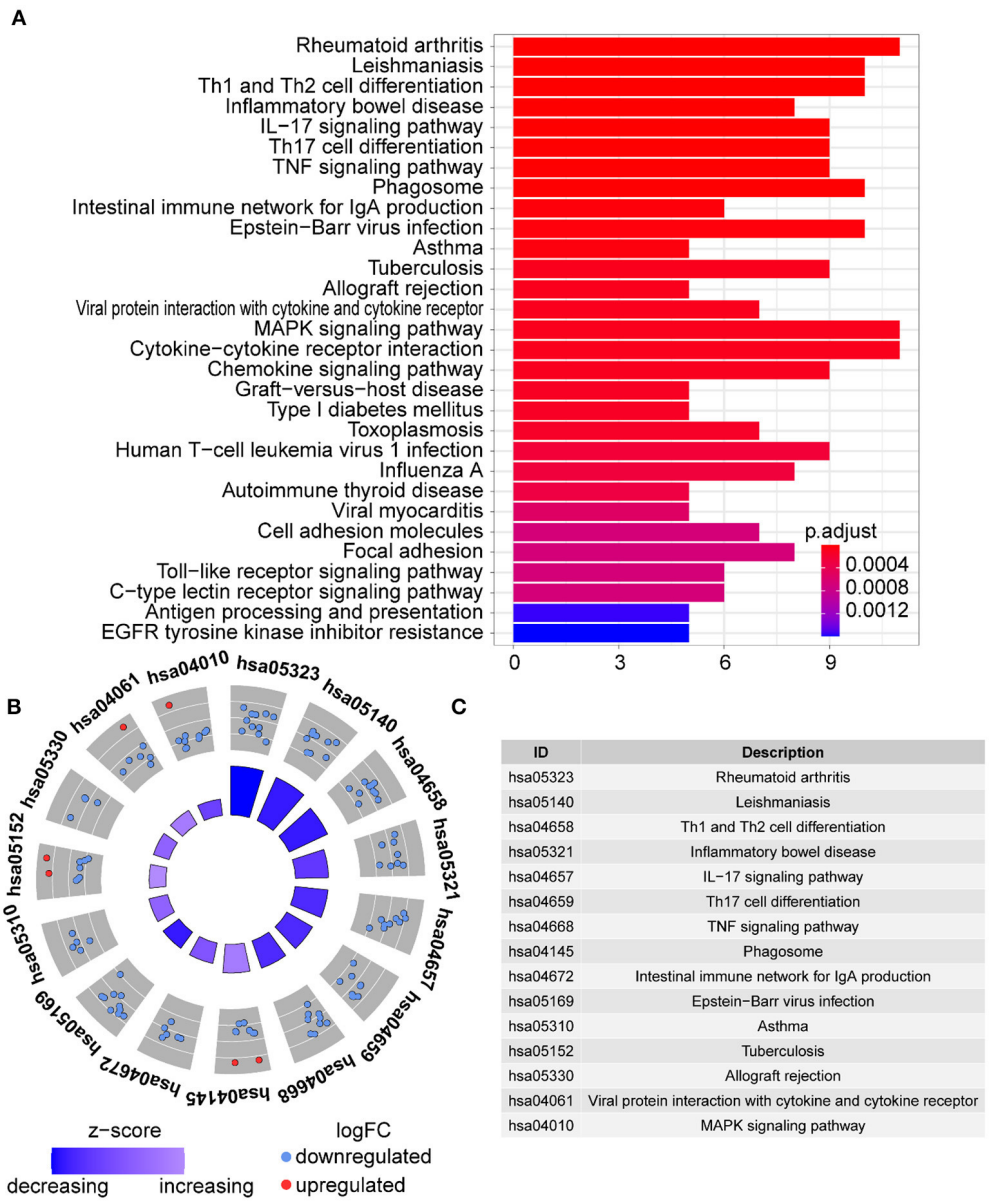
KEGG analysis of the DE-IGs between inflamed and fibrotic livers of BA. **(A)** The 30 most significantly enriched KEGG pathways are displayed in the barplot. The length of the bar represents the number of genes enriched and the color represents the correlation. **(B)** The circle of KEGG enrichment analysis. Each dot in the circle represents a gene, and the outer circle reflects the enrichment of the DE-IGs in different signaling pathways. The inner-circle represents the Z-score, the color depth corresponding to the Z-score. Higher Z-score indicates a higher expression of the DE-IGs in enriched pathways. **(C)** The table annotates KEGG pathways.

In GO term analysis, BP was significantly enriched in the proliferation and function of smooth muscle cell cytokine related terms; CC was mainly enriched in organelles ER, Golgi and related vesicle lumen, and MHC protein complex; and MF was associated with amide and peptide binding, cytokine and chemokine activity, and MHC class II receptor activity (Figure 10). In KEGG pathway analysis, the DE-IGs were mainly enriched in Th1 and Th2 cell differentiation, Th17 cell differentiation, IL-17 signaling, Toll–like receptor signaling, and C–type lectin receptor signaling pathways (Figure 11).

### Immune Cells Infiltrating the BA and Viral Hepatitis B

**Figure 12:**
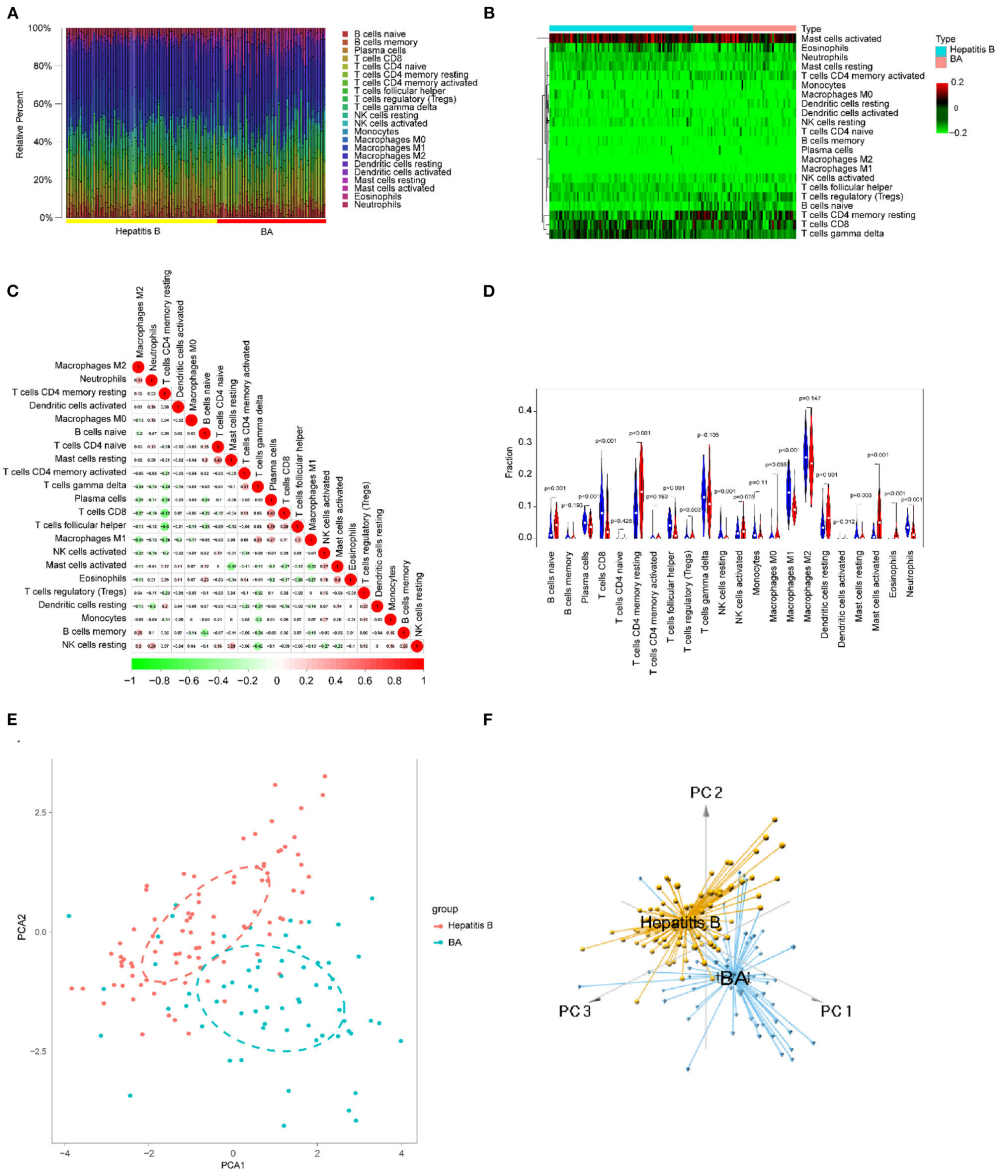
Immune cells infiltrating in BA and viral hepatitis B. **(A)** The barplot reflects the relative content of 22 immune cells in each sample. **(B)** Heatmap of the 22 immune cell proportions. **(C)** Correlation heatmap of 22 immune cells. The size of the colored circles represents the strength of the correlation; red represents a positive correlation and green represents a negative correlation. **(D)** Violin diagram of the proportion of 22 types of immune cells among the different groups. **(E,F)** PCA cluster plot of immune cell infiltration between BA and viral hepatitis B samples.

Using the methods mentioned earlier, the immune cell matrix was obtained and the data were visualized. The gene expression matrix was converted into the immune cell-matrix and combined with the composition and percentages of the immune cells with CIBERSORT. Based on the screening cut-off value, 153 (64 BA and 89 viral hepatitis B) statistically significant immune cell matrices were obtained. The barplot and heatmap reflected the type and amount of immune cell infiltration in each individual (Figures 12A,B). The correlation heatmap indicated that resting CD4^+^ memory T cells had a negatively moderate correlation with CD8^+^ T cells and follicular helper T cells (Figure 12C). The violin map showed that 12 infiltrating immune cells were distributed differently between BA and viral hepatitis B and had statistical a significance (Figure 12D). Contract with viral hepatitis B, naive B cells, resting CD4^+^ memory T cells, resting DCs, and activated mast cells were more broadly distributed, and plasma cells, CD8^+^ T cells, follicular helper T cells, γδT cells, resting NK cells, M1 macrophages, resting mast cells, and neutrophils were less evenly distributed. PCA showed the distinct distribution modes between the BA and viral hepatitis B (Figures 12E,F). The results showed that the two groups could be divided into two independent aspects based on the immune cells infiltrating.

## Discussion

Biliary atresia is a serious biliary system disease with bile duct obstruction caused by a varied etiology, including jaundice and liver failure at early postnatal stages (19, 20), which is more likely to be an anatomical diagnosis than an etiological diagnosis. At present, diagnosing and treating BA are still difficult, and the prognosis is not ideal. The main treatments for BA are Kasai hepatic portoenterostomy (HPE) and liver transplantation (LT) (1, 2). Although some patients with BA underwent Kasai's operation and benefited from it, almost all patients with BA eventually needed to receive liver transplants. There were reasons for the surgical technique, but the essence was that the disease itself was not fundamentally resolved and remained progressive. However, donor liver grafts are rare, and the costs of liver transplantation and postoperative maintenance therapy are costly. Therefore, it is urgent to clarify the etiology to seek new therapeutic options and establish new programs to improve the prognosis of BA.

**Figure 13:**
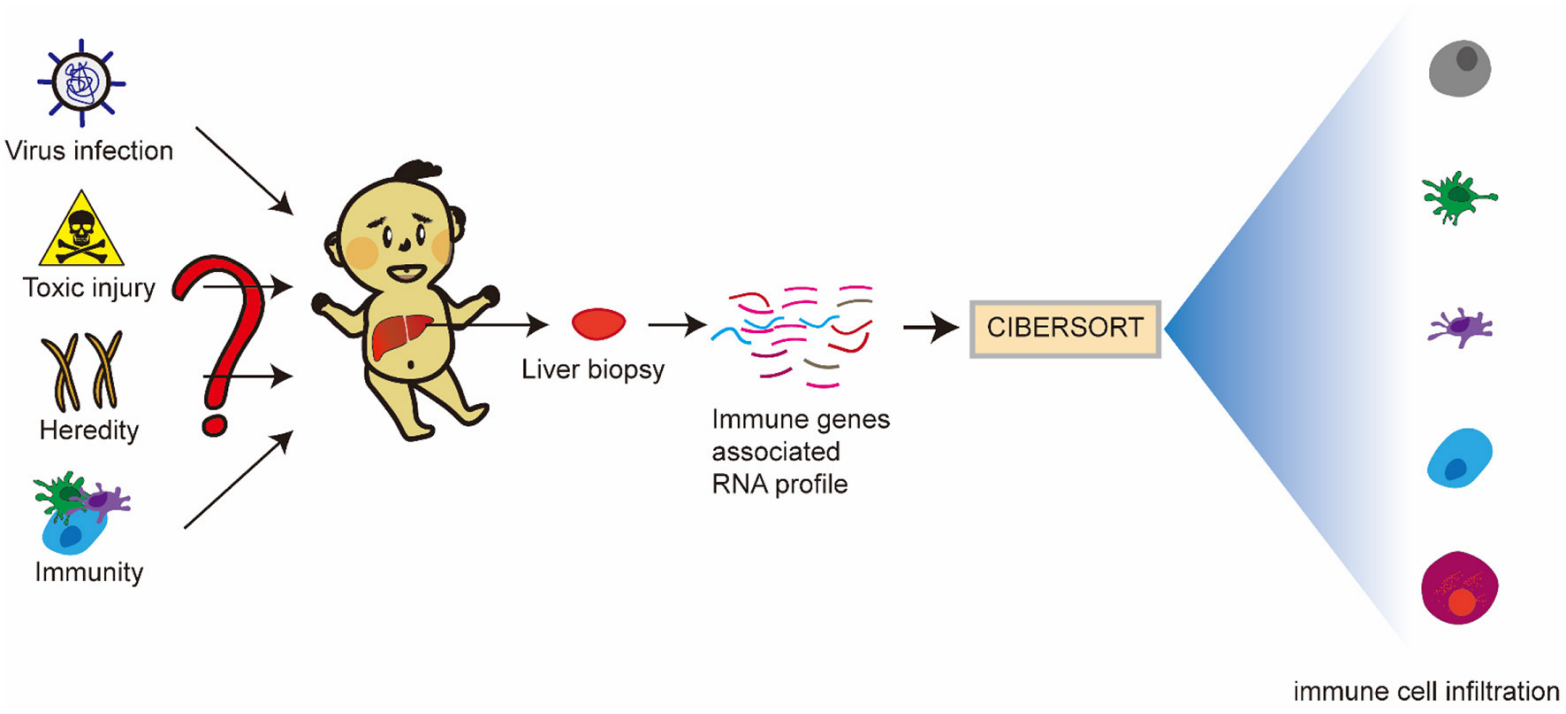
Article hypothesis and ideas. BA may be an immune-related disease mediated by viral infection, toxic injury, and genetics, or even an autoimmune disease. Inherent and specific immunity play an important role in the occurrence and development of BA. DE-IGs in BA livers, normal livers, and hepatitis B livers were analyzed, and the proportion of 22 immune cells in all samples of the dataset was predicted using CIBERSORT software to elucidate the pathogenesis of BA.

The causes and pathogenesis of BA remain unknown, and possible causes include genetic, environmental toxins, viral, or immune factors. Although there is no clear evidence that BA is inherited and identical twins did not develop BA at the same time, alterations in the expression levels of certain genes may cause bile duct cells to be more susceptible to external attack and develop BA (2, 21). Several naturally occurring BA outbreaks in Australian livestock have been associated with the ingestion of unusual plants by pregnant animals under drought conditions, and in essence, may be related to the isoflavones in these plants (21). Infections with viruses, such as Reovirus, rotavirus, and CMV, have also been suggested as potential causes of BA. Mouse models of RRV-induced bile duct injury are often used to study the role of viruses and inflammation in the pathogenesis of bile duct injury. The volume of literature supports the association of viral infection with cytomegalovirus as the initiating event in the pathogenesis of BA, and in most BA children with CMV-DNA and CMV-IgM positivity (2). Although the etiology is unknown, the possible influences now point to a common change in the development of the disease, an abnormality in the immune system. Either a toxic or a viral infection can cause abnormal activation of the immune system, or bile spillage outside the biliary tract can activate the immune system, or the immune system itself may be altered, similar to an autoimmune disease (Figure 13).

Existing studies have shown that immune system disorders are associated with BA. Both innate immunity and specific immunity are involved in BA (3, 9, 13). Therefore, we hope to find the differences between BA and normal livers from the point of view of immune-related genes and immune cell infiltration to understand whether there is a genetic predisposition to BA and the role the immune system plays in the disease.

Comparing BA livers with normal livers, 73 DE-IGs were screened from the GSE46960 and GSE15235 datasets, including 111 BA and 17 donor livers. After that, we found that even in BA, the activated immune genes were different at different stages of BA. Interestingly, DE-IGs were all expressed at low levels in fibrotic livers in patients with BA. Some viruses, such as CMV, have been demonstrated to be associated with BA, but a few studies report that hepadnaviridae, like HBV and HCV, could cause BA, which especially can be transmitted vertically from mothers to babies, especially in China (20, 22, 23). Some studies have reported that HBV and HCV genomes were detected, and HBc and/or HBs' antigens were positive in BA (22, 23). Humoral immunogenicity was insufficient after recombinant HBV vaccination in children with BA (15). Moreover, patients with BA developed HBV infection at a certain time point after liver transplantation, which may be related to postoperative immunosuppressive therapy, but is not a sufficient necessity. Maybe the two just exist at the same time, or there may be some connection between the two. Finally, based on the uncertain association of hepadnaviridae with BA, we collected data of 64 BA and 123 viral hepatitis B from the two datasets GSE46960 and GSE84044 and found 75 DE-IGs.

We performed GO and KEGG analyses of the three differentially expressed genes. The differentially expressed genes of BA and normal liver and the differentially expressed genes of BA and hepatitis B were enriched in most of the same pathways. In GO term analysis, CC was associated with the MHC protein complex, and MF was associated with cytokine and chemokine activity and MHC class II receptor activity. In KEGG pathway analysis, the DE-IGs were mainly enriched in the Th1 and Th2 cell differentiation, Th17 cell differentiation, IL-17 signaling, Toll–like receptor signaling and TNF signaling pathways, and autoimmune diseases.

Toll-like receptors (TLRs) are the commonly reported pattern recognition receptors (PRRs) in humans. Different kinds of immune cells (i.e., monocytes, hepatic macrophages, dendritic cells, and cholangiocytes) express various TLRs on their membranes. TLRs activation induces the production of pro-inflammatory cytokines and chemokines enhancing the participation of effector cells in the immune response (24). In BA liver tissue, increased TLR3 and TLR7 activate an inflammatory cascade by inducing a type 1 interferon response (25, 26). Plasma concentrations for IL-17A and Th17 cell responses in the liver were demonstrated in late phase BA. In experimental BA, γδT cells were the primary cellular source for IL-17A production during early acute inflammation, whereas Th17 responses perpetuate intrahepatic biliary epithelial cells injury in the post-obstructive phase of BA (27).

In fact, the immune factors are not only a part of the immune response but also the embodiment of the function of immune cells. We further analyzed the infiltration of immune cells in the liver to better understand the role of the immune system in BA. CIBERSORT software could not predict the immune infiltration of normal livers. We speculated that this was because the immune infiltration of the normal liver was in a resting state and the immune genes were not activated. Therefore, we only compared the immune infiltration of different pathological types of BA and the immune infiltration of BA and hepatitis B.

Activated mast cells, eosinophils, and neutrophils were more abundant in inflamed livers than in fibrotic livers. In contrast, CD8^+^ T cells and γδT cells were more abundant in fibrotic livers (Figures 8A,B). These results indicated that in BA, the early phase innate immunity is the dominant immune response, and specific immunity is devoted to the development of fibrosis and cirrhosis in the later phase. The correlation heatmap showed the relationship between different immune cells (Figure 8C). All of these immune cells were reported to be associated with BA. As mentioned above, γδ T cells produce IL17, which is required for the inflammation and destruction of the biliary system (27). Macrophages, as effector cells, could produce the reactive oxygen species (ROS), nitric oxide (NO), and lysosomal enzymes to kill bill duct cells (28). Guo et al. found up-regulation of CD8^+^ cytotoxic T-lymphocytes (CTLs) and their costimulatory molecules in patients with BA, indicating a toxic function executed by these CTLs (29). In experimental BA, CD8^+^ T cells were demonstrated to be an effector of cholangiocyte injury. RRV-primed DCs stimulated CD8^+^ T cells interferon-gamma (IFN-γ) production, and Treg-depleted animal model showed the expansion of hepatic CD8^+^ cells and the enhancement of the stimulating ability of hepatic DC cells, which leads to the injury of bile duct epithelial cells more easily (30). The violin map showed that there were more M1 macrophages and resting mast cells in fibrotic livers, which further suggested that these cells may play a promoting role in the process of BA. Macrophages are divided into two subtypes: classical activation, also known as M1, and selectable activation, also known as M2. The evolution of macrophages to different functional subtypes is called polarization. Macrophages activated to the M1 phenotype may be related to biliary epithelial cells injury (9). Promoting the polarization of M1 macrophages to M2 macrophages may be a strategy to alleviate bile duct injury and chronic liver injury after obstruction in BA. The immune infiltration mode of BA was obviously different from that of hepatitis B, and PCA made a clear distinction between the two groups, thus distinguishing the two diseases.

However, there are limitations in this study; the immune system is in a dynamic process of change since its development begins in embryonic life until adulthood, so it is a challenge to study the role played by immunity in BA. Intrahepatic biliary depression jaundice, such as choledochal cysts, is commonly used as a control group for BA, but these diseases themselves cause alterations in the immune cells, in the background of the liver, and therefore donor liver specimens were used as controls in this study. BA is a perinatal disease, although the exact time point of onset is undetermined, and pathology is mostly taken at the time of biliary exploratory surgery, Kasai's surgery, and liver transplantation, so there is a significant period of time to take the specimens. Also, because of ethical constraints, it is difficult to obtain normal liver tissue and achieve perfect age matching. Therefore, it remains to be further elucidated how the pattern of immune cell infiltration differs between normal neonatal and infant livers and normal livers of children or adults. The immune cell infiltration pattern and possible pathogenesis of biliary atresia described in this study is a prediction at the bioinformatics level, but it is also a direction for the next studies. Jun Wang et al. reported that anti-CD20 treatment with rituximab (RTX) could alter BA immune cell function and alleviate the clinical symptoms of BA (31). Although they reported only four cases, it showed that our scenario was scientifically sound. This provided ideas for postoperative adjuvant therapy for Kasai's surgery, potentially extending the interval between the need for postoperative liver transplantation and relieving the pressure of lack of liver source.

## Conclusion

Biliary atresia (BA) is a serious biliary disease in infants. Bile duct cell injury and the loss of intrahepatic and extrahepatic bile ducts may occur during the embryonic period. The main clinical manifestation is jaundice, which is difficult to distinguish from other jaundice diseases. At present, the most effective treatment is surgery, including Kasai's operation and liver transplantation. Based on immune genes and immune cell infiltration, this study reveals the immune characteristics of BA from a global point of view, which provides a new perspective for understanding the pathogenesis of BA and provides a direction for the diagnosis and treatment of BA. We would also like to emphasize that our aim is not to start a new diagnostic paradigm, but to complement the existing diagnostic modalities with a diagnosis of BA at the molecular and cellular level, and to provide some ideas for adjuvant therapy after Kasai's surgery from an immunological perspective to extend the interval between Kasai's surgery and liver transplantation and to relieve the pressure of liver transplantation.

## Data Availability Statement

The datasets presented in this study can be found in online repositories. The names of the repository/repositories and accession number(s) can be found in the article/supplementary material.

## Ethics Statement

The studies involving human participants were reviewed and approved by the Institutional Review Board of Children's Hospital of Chongqing Medical University. Written informed consent to participate in this study was provided by the participants' legal guardian/next of kin.

## Author Contributions

All authors contributed to data analysis, drafting, and revising of the article, gave final approval of the version to be published, and agree to be accountable for all aspects of the work.

## Conflict of Interest

The authors declare that the research was conducted in the absence of any commercial or financial relationships that could be construed as a potential conflict of interest.

## Publisher's Note

All claims expressed in this article are solely those of the authors and do not necessarily represent those of their affiliated organizations, or those of the publisher, the editors and the reviewers. Any product that may be evaluated in this article, or claim that may be made by its manufacturer, is not guaranteed or endorsed by the publisher.
